# Catheter-based Intramyocardial Injection of FGF1 or NRG1-loaded MPs Improves Cardiac Function in a Preclinical Model of Ischemia-Reperfusion

**DOI:** 10.1038/srep25932

**Published:** 2016-05-17

**Authors:** Elisa Garbayo, Juan José Gavira, Manuel Garcia de Yebenes, Beatriz Pelacho, Gloria Abizanda, Hugo Lana, María José Blanco-Prieto, Felipe Prosper

**Affiliations:** 1 Department of Pharmacy and Pharmaceutical Technology, School of Pharmacy, University of Navarra, Pamplona, Spain; 2 Instituto de Investigacion Sanitaria de Navarra (IDISNA), Pamplona, Spain; 3 Hematology, Cardiology and Cell Therapy, Clínica Universidad de Navarra and Foundation for Applied Medical Research, University of Navarra, Pamplona, Spain

## Abstract

Cardiovascular protein therapeutics such as neuregulin (NRG1) and acidic-fibroblast growth factor (FGF1) requires new formulation strategies that allow for sustained bioavailability of the drug in the infarcted myocardium. However, there is no FDA-approved injectable protein delivery platform due to translational concerns about biomaterial administration through cardiac catheters. We therefore sought to evaluate the efficacy of percutaneous intramyocardial injection of poly(lactic-co-glycolic acid) microparticles (MPs) loaded with NRG1 and FGF1 using the NOGA MYOSTAR injection catheter in a porcine model of ischemia-reperfusion. NRG1- and FGF1-loaded MPs were prepared using a multiple emulsion solvent-evaporation technique. Infarcted pigs were treated one week after ischemia-reperfusion with MPs containing NRG1, FGF1 or non-loaded MPs delivered via clinically-translatable percutaneous transendocardial-injection. Three months post-treatment, echocardiography indicated a significant improvement in systolic and diastolic cardiac function. Moreover, improvement in bipolar voltage and decrease in transmural infarct progression was demonstrated by electromechanical NOGA-mapping. Functional benefit was associated with an increase in myocardial vascularization and remodeling. These findings in a large animal model of ischemia-reperfusion demonstrate the feasibility and efficacy of using MPs as a delivery system for growth factors and provide strong evidence to move forward with clinical studies using therapeutic proteins combined with catheter-compatible biomaterials.

Heart failure remains the leading global cause of death^1^. Growing evidence indicates that growth factor therapy is a promising approach to treat myocardial infarction (MI)^2^,^3^. Therapeutic proteins, including neuregulin-1 (NRG1), acidic-fibroblast growth factor (FGF1), vascular endothelial growth factor and erythropoietin have been implicated in the mechanism of cardiac repair after MI^3^,^4^,^5^,^6^. However, despite several compelling preclinical and initial clinical studies^7^,^8^,^9^, double-blinded clinical trials with large cohorts of patients have failed to validate the efficacy of protein therapy in MI patients^10^,^11^,^12^,^13^,^14^. Limited stability and rapid degradation after administration are critical challenges that may hamper the translation of therapeutic proteins into widespread clinical use. Proteins require new formulation strategies that allow for sustained bioavailability of the protein locally in the infarcted myocardium. The combination of injectable biomaterials with growth factors represents a key strategy able to address shortcomings of protein therapy for cardiac regeneration. However, although extensive research has been performed in this area, there is no FDA-approved injectable protein delivery platform for MI treatment at present, due to translational concerns related to biomaterial administration through cardiac catheters.

Different biomaterials have been investigated for cardiac regeneration^15^,^16^,^17^. Of particular interest in the field of regenerative medicine are synthetic polymers like the polyesters poly(lactic-co-glycolic acid) (PLGA), which have reached FDA approval for clinical application in tissue repair^18^ with a demonstrated track record as vehicles for protein delivery. Significant research has been carried out on the development of bioresorbable stent scaffolds^19^,^20^,^21^ and drug delivery systems^22^,^23^ using PLGA polymer for heart tissue engineering applications. Interestingly, PLGA can be shaped/processed into delivery systems like microparticles (MPs), which can be injected through cardiac catheters allowing controlled local delivery of proteins directly in relevant areas of the heart^24^,^25^,^26^. Recently, we demonstrated the benefit of incorporating NRG1 and FGF1 within bioresorbable PLGA-MPs that can generate sustained growth factor levels in the ischemic myocardium in a rat MI model, leading to induction of tissue revascularization, activation of endogenous regeneration and eventually improving heart function^26^. PLGA-MPs were prepared by a multiple emulsion solvent-evaporation technique using the Total Recirculation One-Machine System (TROMS). This technology produces very homogeneous batches on a semi-industrial scale, which is of great interest for future industrial manufacturing.

The goal of this study was to scale up our previous studies^26^ into a clinically-relevant preclinical porcine model of ischemia-reperfusion in order to demonstrate the feasibility and efficacy of percutaneous intramyocardial delivery of PLGA-MPs loaded with NRG1 and FGF1 using the NOGA MYOSTAR injection catheter. Notably, the percutaneous delivery of growth factor loaded MPs through the catheter-based NOGA navigating system achieved a sustained growth factor release in the MI region and a significant recovery of cardiac function associated with therapeutic neovascularization and remodeling.

## Results

### Preparation and characterization of injectable growth factor loaded MPs

**Figure 1:**
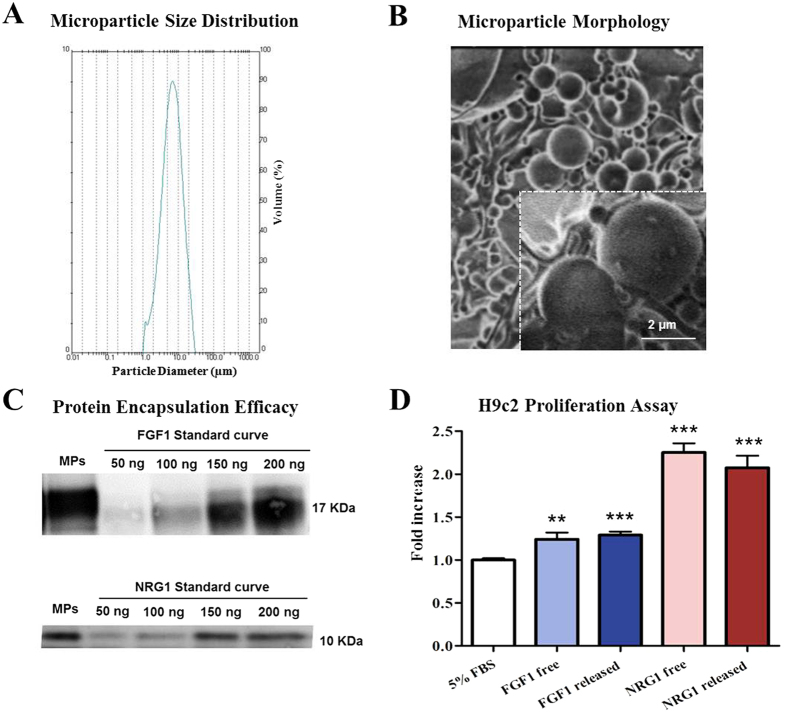
Characterization of injectable MPs loaded with FGF1 and NRG1. (**A**) MP size distribution. (**B**) Scanning electron microscopy images showing the morphology of MPs. (**C**) Encapsulation efficiency. Western blot bands corresponding to FGF1/NRG1 standard curve and FGF1/NRG1 encapsulated in MPs (**D**) *In vitro* effect of MP-released cytokines on heart-derived cells. H9c2 cells were cultured in the presence of either MP-released or free FGF1 and NRG1. Values are the mean fold-increase in cell number over a 72-h period ± SEM from three independent experiments. (One–way ANOVA with a post-hoc Tukey’s multiple comparison test; **p < 0.01 ***p < 0.001).

FGF1 and NRG1 were successfully encapsulated in PLGA MPs prepared by multiple emulsion solvent evaporation technique using the TROMS. The mean particle size measured by laser diffractometry was 7.2 ± 1.9 μm, with a range of particle size from 0.5 to 35 μm, which is compatible with an intramyocardial administration using the 27 G NOGA catheter^27^,^28^ (Fig. 1A). Scanning electron microscopy (SEM) analysis showed that PLGA MPs had a spherical shape with a smooth surface (Fig. 1B). One purpose of the study was to prepare MPs loaded with at least 250 μg/100 mg polymer to allow dose scale up according to body weight in transition from small to large animal models of MI. To this aim, the amount of therapeutic protein loaded into MPs (100 mg) was therefore increased to 300 μg. Growth factors were efficiently encapsulated, reaching values of 84 ± 15.5% for FGF1 and 83.5 ± 13.2% for NRG1 determined by western blot (Fig. 1C). The total amount of loaded FGF1 and NRG1 was 252.1 ± 46.5 and 250.5 ± 39.7 μg/100 mg polymer respectively, suitable for *in vivo* studies in minipigs. Both formulations showed low initial protein burst release *in vitro* (*i.e*. ~12.6% of NRG1 and ~10% of FGF1 were released during the first 15 hours) as previously reported by our group for this kind of formulation^26^. The bioactivity of the cytokines released from the MPs was evaluated by the induction of H9c2 cardiomyocyte proliferation. The rate of cell proliferation was significantly increased 1.3 and 2.1 fold when stimulated with FGF1 and NRG1 released from MPs respectively (p < 0.001 vs. control). Proliferation rates of free-cytokine treated-cells were similar to those obtained with the cytokines released by MPs, indicating that both cytokines remained bioactive after microencapsulation and release, and demonstrating that the developed delivery system allowed the maintenance of protein stability during manufacture and release (Fig. 1D).

### FGF1 and NRG1-MPs improves cardiac function after ischemia-reperfusion in pigs

We then tested our hypothesis that injection of FGF1 or NRG-1 MPs would improve cardiac function after ischemia-reperfusion in a large animal model of MI. Seven days after ischemia-reperfusion animals were divided in three groups and treated with transendocardial injections of FGF1-MPs (n = 6), NRG1-MPs (n = 5) or control-MPs (non-loaded) (n = 6) using the Myostar catheter. The mean number of injections per animal was 27.7 ± 0.9, 27 ± 1.9 and 27.3 ± 3.5 respectively. The amount of MPs was 79.3 mg, 79.8 mg and 79.8 mg respectively. The volume administered in each injection was 0.3 mL. MP suspension (±10 mg MPs/mL) showed good injectability through the NOGA-guided MyoStar catheter equipped with a 27 G needle. Particle aggregation, catheter clogging or needle blockage during the administration of the MP suspension was not observed. During transendocardial injection, 2 animals experienced ventricular tachycardia and required cardioversion. No other side effects (arrhythmias or other cardiac events) were documented before or after the procedure.

**Figure 2:**
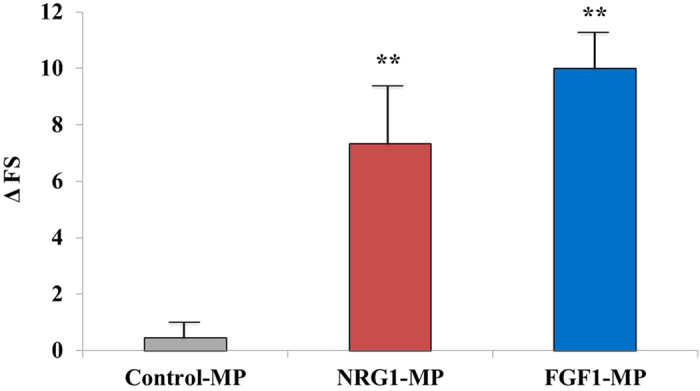
Echocardiographic data shows improvement in systolic function after treatment with FGF1 and NRG1 loaded MPs. FS was assessed by echocardiography in minipigs 3 months after NRG1-MPs or FGF1-MPs injection. Data are expressed as mean ± SEM (One–way ANOVA with a post-hoc Tukey’s multiple comparison test; **p  < 0.01).

Temporal coronary occlusion followed by reperfusion caused a marked decrease in fractional shortening (FS) at day 7 (38.6 ± 3.8% at baseline versus 19.4 ± 3.5 before implant; p < 0.0001), which did not significantly differ among groups (p = 0.56). Measurement of cardiac function was evaluated 3 months after NOGA-guided transendocardial MP injection. While no significant changes were found in the FS in the control group 3 months after implant (Control-MPs: 19.1 ± 2.1), transplantation of NRG1-MPs or FGF1-MPs was associated with a statistically significant benefit in cardiac contractility as shown by higher FS (FGF1-MPs: 28.2 ± 6.1, NRG1-MPs: 27.9 ± 6.1) (Fig. 2).

Additionally, FGF1-MP and NRG1-MP injection prevented the deterioration of ventricle geometry, as indicated by the decrease in end-systolic and end-diastolic diameters (Table 1) in comparison with control animals, where a progressive worsening of the ventricular diameters was observed. Altogether, these results demonstrate a beneficial effect of the MPs, inducing adequate remodeling of the heart.

**Figure 3:**
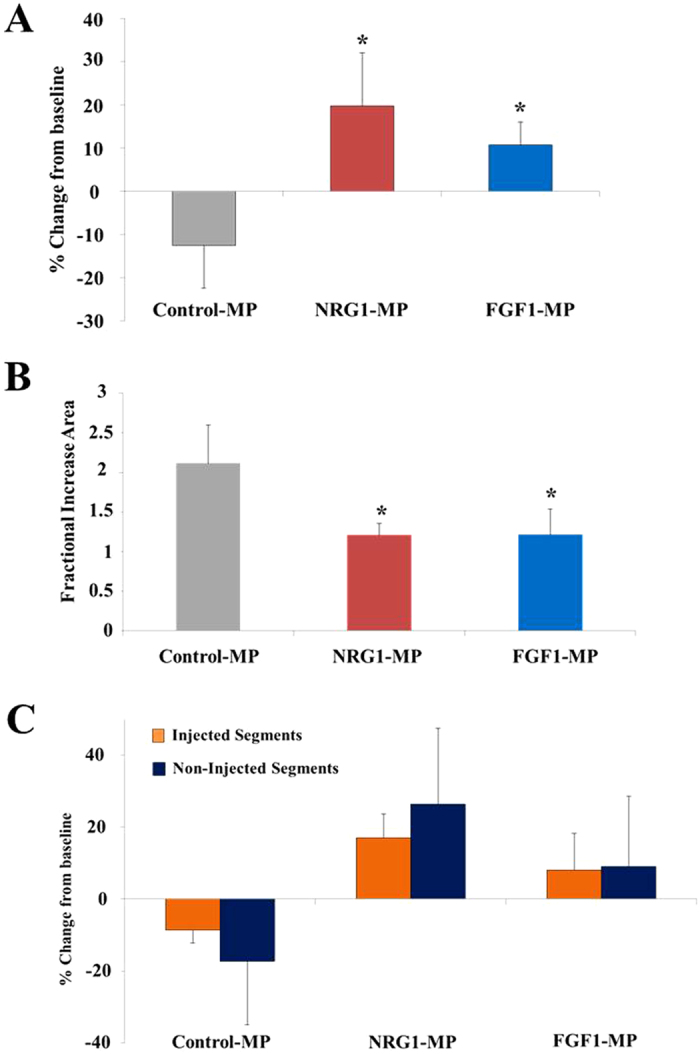
Transcatheter based, NOGA mapping guided intramyocardial injection of growth factor loaded-MPs in pigs. (**A**) Bipolar changes three months after implantation in comparison with baseline levels. (**B**) Infarct expansion calculated as the increase in the area with bipolar voltage values lower than 0.8 mV 3 months after treatment. (**C**) Bipolar changes three months after injection comparing segments in which particles were directly injected or non-injected. Data in A, B and C are means ± SEM. (One–way ANOVA with a post-hoc Tukey’s multiple comparison test; *p < 0.05).

Electromechanical NOGA data were collected pre-injection and before sacrifice in control-MPs (n = 3), NRG1-MPs (n = 5) and FGF1-MPs (n = 4) treated animals. Bipolar voltages were analyzed in 9 bulls-eye segments used to calculate the change in tissue viability and transmural infarct dimensions over the course of the experiment, as this method has been demonstrated to accurately predict the transmural extent of myocardial infarction^29^. Bipolar voltage measurements at time of injection and after 3 months revealed an overall reduction of −12.5 ± 9.9% from baseline values in control pigs and a significant increase in NRG1-MPs (+19.8 ± 12.3%) and FGF1-MPs (+10.7 ± 5.2%) treated animals (p < 0.05) (Fig. 3A).

**Figure 4:**
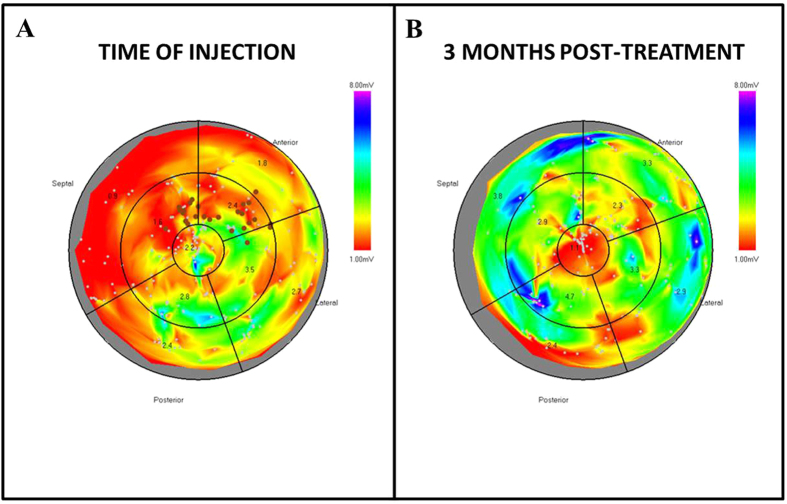
Representative bipolar area NOGA maps taken at time of injection (**A**) and euthanasia (**B**) of a NRG1-MP treated animal. The mapping points are shown by white dots. Dark dots in A, represent the injection sites.

Moreover, infarct areas were extracted from NOGA maps in order to evaluate the effect of MP injection on the extent of the infarcted area. Areas with bipolar voltage values lower than 0.8 mV were considered transmural scar, as previously described^30^. In control-MP treated animals we observed an average fractional increase of 2.1 ± 0.5 indicating a doubling of the transmural infarct areas. However, in animals treated with NRG1-MPs and FGF1-MPs the infarct size increased only marginally (1.2 ± 0.1 and 1.2 ± 0.3) (Fig. 3B). Differences between control-MP treated animals and NRG1-MP or FGF1-MP treated pigs were statistically significant (p < 0.05). When injected and non-injected segments were analyzed separately and compared between control and animals treated with growth factors loaded MPs, changes in bipolar voltages in non-injected segments showed the same pattern as changes observed when only injected segments were analyzed (Fig. 3C), suggesting growth factor diffusion to other heart areas. Representative bipolar map changes of a NRG1-MP treated animal at baseline and before sacrifice are illustrated in Fig. 4. Collectively, these results indicate that the injection of NRG1 and FGF1-MPs improves cardiac function after MI.

### FGF1 and NRG1-MPs promote heart tissue revascularization

**Figure 5:**
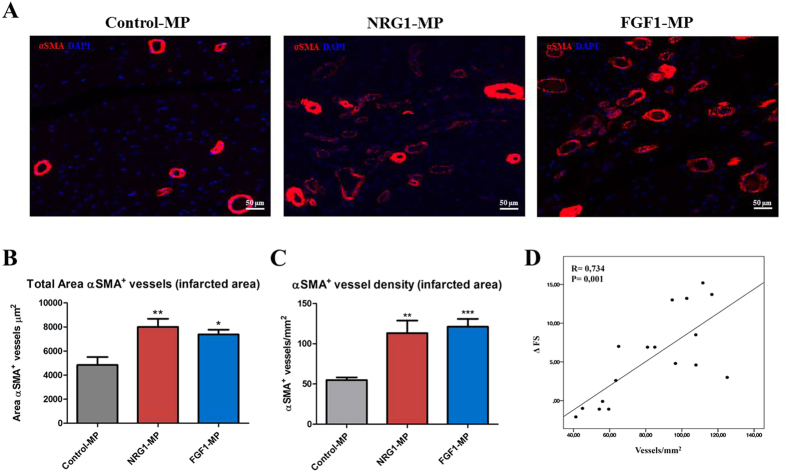
FGF1 and NRG1 released from MPs in the ischemic miocardium lead to angiogenic effects. (**A**) Representative images showing the infarcted/peri-infarcted area three months after treatment stained with antibodies against blood vessels (α-SMA^+^) and nuclei (DAPI) in sections of each group of animals. Quantification of α-SMA total area (**B**) and vessel density (**C**) in infarcted and peri-infarcted zones 3 months after growth factor loaded MP or control-MP injection. Data are means ± SEM. (One–way ANOVA with a post-hoc Tukey’s multiple comparison test; *p < 0.05 **p < 0.01 ***p < 0.001). (**D**) Correlation between functional echocardiographic parameters and α-SMA vessel density (Pearson Correlation analysis R =  0.734; p =  0.001).

No adverse cellular reactions or signs suggestive of localized toxicity from the MPs were observed during the histological evaluation of the hearts. We then determined the effect of MPs injection on vasculogenesis and analyzed whether functional efficacy was accompanied by increased revascularization. Three months after treatment, a significant increase in vascularization was detected in the ischemic zone at the arteriolar/arteries level (Fig. 5A). The α-smooth muscle actin (αSMA)^+^ vessel area was significantly increased after the administration of any of the cytokine-loaded MPs in comparison with the control-MPs (Control-MPs: 4838.56 ± 655.6 μm^2^, NRG1-MPs: 7993.76 ± 675.72 μm^2^; *P* < 0.01, FGF1-MPs: 7380.91 ± 387.73 μm^2^; *P* < 0.05) (Fig. 5B). Additionally, the αSMA^+^ vessel density was significantly greater in animals treated with FGF1-MPs or NRG1-MPs: (Control-MPs: 54.92 ± 3.32 vessels/mm^2^, NRG1-MPs: 113.22 ± 15.46 vessels/mm^2^; *P* < 0.01, FGF1-MPs: 121.15 ± 9.71 vessels/mm^2^; *P* < 0.001) (Fig. 5C). Thus, transplantation of FGF1 and NRG1-MPs promoted heart tissue vascularization. Furthermore, a significant positive correlation between FS and vasculogenesis was observed (R = 0.734; p = 0.001), suggesting that cytokine loaded MPs may contribute to improvement in cardiac function by increasing the vasculogenesis in the infarct tissue (Fig. 5D).

### NRG1-MPs reverse cardiac fibrosis

**Figure 6:**
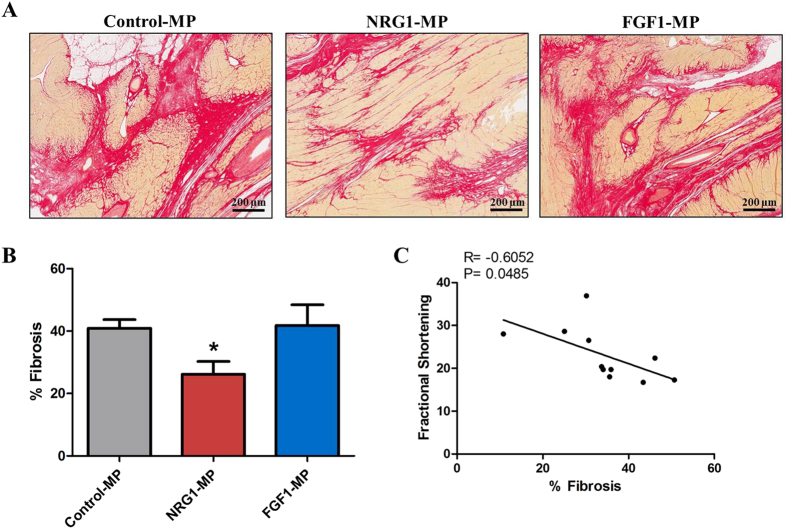
Effect of FGF1 and NRG1-MP on cardiac fibrosis. (**A**) Representative images showing the infarcted area three months after treatment stained with Sirius Red in sections of each group of animals. (**B**) Quantification of collagen deposition in the infarcted zones 3 months after growth factor loaded MP or control-MP injection. Data are means ± SEM. (Kolmogorov-Smirnov test showed no Gaussian distribution and the non-parametric Kruskal Wallis test was used; *p < 0.05). (**C**) Correlation between functional echocardiographic parameters and % fibrosis between MRG1-MP and Control-MP (Pearson Correlation analysis R = −0.6052; p = 0.0485).

Finally, to ascertain the effect of growth factor-loaded MPs in cardiac fibrosis and analyze whether cardiac function improvement was associated with a lower degree of fibrosis, we quantified the total collagen content in animals receiving NRG1- or FGF1-MPs (Fig. 6). It was observed that NRG1-MPs reversed cardiac fibrosis in the infarcted area (Fig. 6A). Quantification of collagen deposition shows that the degree of fibrosis in the infarct zone was significantly diminished in the animals treated with NRG1-MPs in comparison with the control group three months after treatment. On the other hand, a significant effect on tissue remodeling was not detected in animals treated with FGF1-MPs (% Fibrosis: Control-MPs: 40.9 ± 2.8%, NRG1-MPs: 26.2 ± 4.5%; *P* < 0.05, FGF1-MPs: 41.8 ± 6.6%,) (Fig. 6B). Notably, we observed a significant negative correlation between FS and the degree of fibrosis between NRG1-MP and Control-MP treated groups (R = −0.6052; p = 0.0485) suggesting that NRG1-MP may improve cardiac function by attenuating cardiac fibrosis in the ischemic area.

## Discussion

The present study demonstrates the feasibility and efficacy of MPs as an injectable protein delivery biomaterial for treating MI through minimally invasive approaches in a clinically relevant model of ischemia-reperfusion. Our findings further show that FGF1 or NRG1 administration via MPs in minipigs has therapeutic effects, supporting the role of this strategy for administration of therapeutic proteins to repair injured heart tissue.

Cardiac biomaterials are a promising approach for MI treatment^31^. Advanced delivery methods combining biomaterials with therapeutic growth factors are needed to maximize protein therapeutic potential, reduce deleterious effects and aid clinical translation^2^. Among drug delivery systems, MPs are able to provide sustained protein release and a decrease in drug dosage, holding great promise in cardiac tissue engineering. These drug delivery systems can be injected through cardiac catheters in a minimally invasive manner, which is extremely important for a faster clinical translation. Thus, examining MP safety and efficacy is critical for the development of new vehicle that can be used in combination with protein-based approaches for MI treatment. In this regard, we previously characterized the cellular and molecular mechanism of FGF1 and NRG1-MPs as well as its efficacy on small animal models of acute MI^26^. Our previous work indicated that treatment with FGF1 and NRG1-MPs in a rat model of acute MI induced an improvement in cardiac function which was associated with cardiomyocyte proliferation, progenitor cell recruitment, increased angiogenesis and decreased fibrosis^26^. Based on these results, in the current study we used a clinically relevant large-animal model of MI to examine the translational potential of this therapy. In the current paper, we not only confirm our previous results, demonstrating the therapeutic efficacy of combining MP-based delivery systems with FGF1 or NRG1 in a pig MI model^24^,^25^,^26^, but also further demonstrate for the first time the feasibility of MPs as an injectable material for treating MI through minimally invasive approaches, proving its potential for clinical application.

Among the strategies aimed at improving protein therapy, the local administration of growth factors within a biomaterial-based delivery system represents an attractive therapeutic strategy. Numerous studies have already shown that injectable protein-releasing biomaterials are able to improve cardiac function in small animal models of MI^15^. Among them, only hydrogels and nanofibers co-delivered with therapeutic proteins have demonstrated myocardial repair and regeneration in large animal models of MI^32^,^33^,^34^. However, most of these strategies were implanted using an open surgical method, since very few materials can be administered using minimally invasive procedures. In addition, technical difficulties related to hydrogel injection using existing catheter technology, such as rapid gelation during administration or needle blockage due to high hydrogel viscosity, have been reported^35^. In other cases, hydrogels require mixing and incorporation of crosslinking agents prior to injection, which hinders their administration via cardiac catheters. Regarding peptide nanofibers, they need to be optimized to be delivered via catheter to facilitate their translation to clinical practice. Alternatively, as shown in our study, PLGA-MPs may be superior to other drug delivery devices such as cardiac patches, membranes, or nanofibers owing to their injectability through a NOGA guided catheter, as well as their potential for targeting different areas of the heart. Here we show that cytokine-loaded MPs preserved cardiac function post-MI, and also demonstrate that MPs are deliverable via cardiac catheter. MPs were successfully injected in the porcine infarcted myocardium without clogging the catheter or blocking the needle, which shows that this protein delivery biomaterial is clinically relevant for percutaneous injection in the heart.

As demonstrated in the present study, a crucial aspect of biomaterial clinical translation is the delivery method. NOGA technology has several advantages, as it allows for guided, direct intramyocardial injection, which increases biomaterial retention and does not require access to the coronary vessels, reducing the risk of embolization^36^. In our study, no serious adverse events were observed during MP administration, which might indicate the safety of this delivery method. The mapping technology also facilitates the distinction between viable, non-viable, stunned or hibernating myocardium, facilitating targeting to ischemic and peri-ischemic myocardial regions in patients with chronic myocardial ischemia, reducing the likelihood of systemic toxicity of the injected substance due to off-target effects. In the present study, cardiac function was significantly improved after treatment with growth factor loaded MPs as measured by bipolar electrical changes, a proven measurement of infarct transmurality^29^, which was associated with improved contractibility, adequate heart remodeling and increased vascular density. The lack of differences in unipolar voltage potential between the different groups is not surprising, as this parameter might be beneficial for identifying subendocardial scars, but does not determine the electrical status of the transmural myocardium^37^.

Many growth factors have been explored for ischemic heart disease treatment based on their capacity to induce angiogenesis, recruit stem and progenitor cells, or contribute to cardiomyocyte proliferation^2^,^3^. The decision to encapsulate NRG1 was based on previous studies implicating this cytokine in cardiac development, but also in the myocardium physiology, from angiogenesis induction to cardiomyocyte proliferation and cardiac progenitor cell recruitment^4^,^6^,^38^. On the other hand, from the large family of fibroblast growth factors, we focused on FGF1 on the basis of its capacity to stimulate angiogenesis and cardiomyocyte mitosis, and to restore cardiac function after MI^39^. Both PLGA-MP and cytokine dose represent an important aspect for this therapy to be successful. Based on studies from our group that have demonstrated in a rat MI model that PLGA particles remain in the heart 3 months after direct intramyocardial injection^40^ and that the bioactive protein is released in a controlled manner at least for twelve weeks^41^, a dose of 200 μg of cytokine per animal was used. We have previously demonstrated that a short cytokine stimulus, if it is not sustained, is not able to reduce infarct size or to induce therapeutic angiogenesis in a rat MI model, owing to the short half-life of proteins^24^. In support of this, in many of the studies involving the development of drug delivery systems to increase the release and the half-life of the growth factor, controls with the free cytokine have led to lack of efficacy^42^,^43^. What is more, the use of free growth factors in MI phase II clinical trials has been associated with limited success^10^,^11^,^12^,^13^,^14^. For all these reasons, the injection of the free proteins was not examined in the present work. Here, we show that the transendocardial injection of FGF1 or NRG1-MPs post-MI in minipigs induces heart tissue revascularization, proving that FGF1/NRG1 are very potent angiogenic factors and confirming our previous work on a rat MI model^26^. Moreover, the positive correlation between left ventricular ejection fraction (LVEF) and vasculogenesis indicate that cytokine loaded MPs may improve heart function through enhancement of angiogenesis in the core infarct tissue. Similarly, most of the examined biomaterials that brought about an improvement in cardiac function displayed neoangiogenesis^33^,^44^,^45^. The sustained release of NRG1 or FGF1 from MPs may provide a prolonged and effective angiogenic stimulus to the ischemic heart, something that was not achieved in clinical trials with pro-angiogenic factors, and which may explain the disappointing results obtained^46^. Thus, the controlled delivery of FGF1 and NRG1 from MPs may initiate healing at the site of the injection, increasing new vessel formation, restoring the blood supply to the infarcted area, and in consequence, inducing an adequate heart remodeling process. Additionally, NRG1-MPs were also able to reverse cardiac fibrosis, thus affording functional improvement, as previously reported by our group and by others^26^,^47^,^48^. Our data might also suggest that the efficacy of FGF1-MPs is not directly associated with prevention of fibrosis. It was shown previously that FGFs play distinct roles in cardiac remodeling^49^. While FGF16 and FGF21 may prevent cardiac hypertrophy and fibrosis, FGF2 and FGF23 display the opposite effect^49^. However, the role of FGF1 on cardiac fibrosis is less known. Based on our findings, we speculate that the improvement in cardiac function observed in FGF1-MP treated minipigs was not mediated by decreased fibrosis, but rather by the potent angiogenic effect of FGF1.

Limited studies have investigated the impact of biomaterials on cardiac electrophysiology, a concern raised by stem cell injection^50^. In our study, MPs were safely injected without inducing arrhythmia. The small size of MPs and the spread of the material through the tissue are unlikely to alter cardiac electrophysiology. These results are in agreement with recent experiments by Singelyn *et al*.^51^, Seif-Naraghi *et al*.^52^ and Lin *et al*.^53^ reporting that both the injection of hydrogels derived from decellularized ventricular extracellular matrix and peptide nanofibers do not lead to induced arrhythmogenesis. Biomaterial location and distribution in the viable tissue might be also an important factor which determines the production of arrhythmias, and this should be further investigated.

Finally, from a translational perspective, the clinical development of these protein delivery MPs should be relatively straightforward since: 1) PLGA has already been approved for clinical use, 2) the MP production method is easily reproducible and applicable on a semi-industrial scale and 3) implementation of GMP conditions, homogeneity between batches and the possibility to reduce contamination are facilitated using TROMS technique since human intervention is minimized during MP production.

In summary, the findings of this work may translate into an improved MI therapeutic strategy with the potential to improve life expectancy and quality of life in patients with MI, and to reduce costs for the healthcare system.

## Conclusion

These data provide compelling evidence that the percutaneous intramyocardial injection of MPs loaded with FGF1 or NRG1 is clinically feasible and has a therapeutic effect in a large animal model of MI, indicating that catheter delivery of MPs might pave the way towards clinical translation of cardiac regenerative medicine for MI.

## Methods

### Injectable growth factor-loaded PLGA MPs preparation and characterization

FGF1 and NRG1 were separately encapsulated into PLGA MPs through a multiple emulsion solvent-evaporation method using the TROMS^26^. Briefly, 100 mg of PLGA dissolved in 4 mL of dichloromethane/acetone was injected into the inner aqueous phase containing 300 μg of growth factor, 5 mg of human serum albumin (HSA) (Sigma-Aldrich), and 5 μl of poly ethylene glycol 400 (PEG 400) dissolved in 200 μl of phosphate-buffered saline. The inner emulsion was recirculated through the system to become homogenous and injected into the outer aqueous phase composed of 20 mL of 0.5% w/v polyvinyl alcohol (PVA) to obtain the multiple emulsion. After solvent evaporation and polymer precipitation, particles were formed. MPs were washed and lyophilized without cryoprotective agents. Non-loaded MPs, abbreviated control-MPs, were prepared similarly to loaded ones, but without growth factors. MPs were characterized as previously described^26^. Particle size and size distribution were measured by laser diffractometry using a Mastersizer^26^. Particle morphology was characterized by SEM. The amount of growth factors encapsulated in the MPs was determined by dissolving 5 mg of lyophilized loaded MPs in 1 mL of dimethyl sulfoxide (DMSO) followed by quantification using western blot assay. SDS-PAGE was performed onto 12% polyacrylamide gels and after electrophoresis the proteins were transferred onto nitrocellulose membranes. After 1 h blocking with 5% nonfat dried milk in Tris-buffered saline (TBS) plus 0.05% Tween 20, nitrocellulose sheets were incubated overnight at 4 °C with primary antibodies against FGF1 (Abcam Ab-9588 diluted 1:2000) or NRG1 (Santa Cruz SC-1793 diluted 1:50). The binding of primary antibodies was performed by incubating membranes with horseradish peroxidase (HRP)-conjugated anti-rabbit secondary antibody (diluted 1:2000). Immunoreactive bands were, after several washes, visualized using LumiLight Plus western blotting substrate (Roche Diagnostics). Quantitative analysis of MP-extracted growth factor bands was performed by densitometry using ImageQuant software. Sample values were quantified using a standard curve. For the *in vitro* release studies, MPs (1 mg, n = 3) were resuspended by vortexing in 0.5 ml of PBS, pH 7.4 containing 0.1% bovine serum albumin and 0.02% w/w sodium azide. Incubation took place in rotating vials at 37 °C. After 15 hours, samples were centrifuged at 25,000 × g, for 15 min and the amount of drug released was determined by western blot, as described above. The bioactivity of FGF1 and NRG1 released from the MP during the first 15 hours was evaluated *in vitro* by determining H9c2 cardiomyocyte proliferative capacity following growth factor treatment. H9c2 cells obtained from embryonic BD1X rat heart tissue were cultured in DMEM medium supplemented with 10% foetal bovine serum (FBS), 1% glutamine, and 1% penicillin/streptomycin at 37 °C under 5% CO_2_/95% air. Cells were sub-cultured when 60% confluence was achieved. Routine testing confirmed that H9c2 cells were free of mycoplasma during the entire study period. In order to quantify cell proliferation after growth factor stimulation, cells were seeded in 96-well tissue culture plates at a density of 2 × 10^3^ cells/well. After 24 h, medium was removed and H9c2 cells were incubated with 150 ng/mL of growth factor released from MPs over 15 h, which had previously been quantified by western blot, with 150 ng/mL of free growth factors or medium alone as control. Culture medium supplementation was modified for these experiments by reducing the FBS to 5% in the culture medium. Treatments were removed every day, and fresh treatment was added to the cells. After 3 days of treatments, the number of viable cells was determined by MTT assay. The experiment was replicated three times.

### Myocardial infarction porcine model

**Figure 7:**
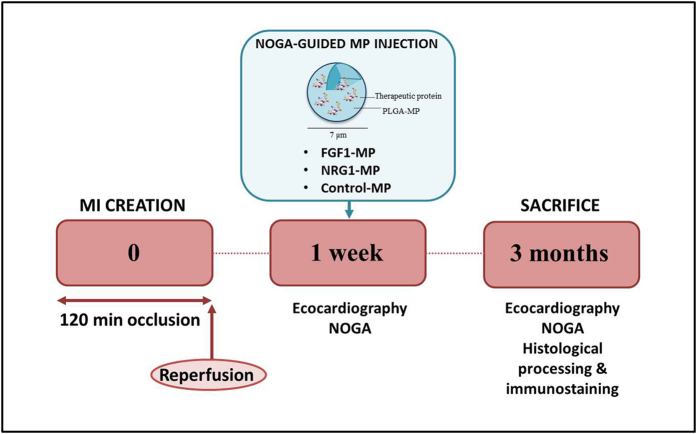
Schematic study design. Adult pigs underwent MI induction by coronary artery occlusion followed by reperfusion. One week later surviving animals underwent electrical mapping followed by intramyocardially injection of control-MPs, NRG1-MPs and FGF1-MPs using a NOGA catheter. Three months later heart electromapping was assessed before sacrifice.

Animal experiments were approved by the Institutional Animal Care and Use Committee of the University of Navarra and performed according to the requirements demanded by the EU legislation. Figure 7 shows the experimental design.

A total of 17 Göttingen minipigs (8 males and 9 females, age between 12 and 24 months at entry in the study, 60–80 Kg) were procured from our GLP accredited breeding center at the University of Navarra and enrolled in the study. MI (ischemia-reperfusion) was induced as previously described^44^,^54^,^55^. A 7-Fr coronary artery-guiding catheter was placed within the ostium under fluoroscopic guidance using a mobile C-arm (Powermobil, Siemens) MI was produced by occluding the left anterior descending coronary artery with a balloon catheter 2.5 just below the second diagonal branch. Temporary and complete occlusion was performed by balloon dilatation (8 atm) and maintained for 120 min, followed by reperfusion, as demonstrated by coronary angiography and ST segment elevation and reversion in the electrocardiogram.

### Echocardiographic assessment

Transthoracic two-dimensional echocardiography was assessed using a Sonos 5500 ultrasound system (Philips) and a 3 MHz linear array transducer. Left ventricular remodeling was determined by measuring end systolic and end diastolic volumes and diameters, according to the American Society of Echocardiography. Diastolic function was assessed by mitral filling pulse doppler and mitral annulus tissue doppler. FS was determined according to Teicholz^56^ in parasternal short axis, due to the unreliability of the 4-camera approach in swine^57^,^58^. Electrocardiogram and echocardiograph measures were taken at different points (baseline, pre-implant and sacrifice) by two investigators blinded to the type of treatment.

### NOGA-guided catheter delivery of growth factor-loaded MPs

Only animals in which a region of MI could be detected by echocardiography and a significant reduction in FS (more than 20% reduction from baseline) were included in the study. Thus, 17 minipigs were implanted with growth factor-loaded MPs using a NOGA-guided MyoStar catheter for intra-myocardial transplantation by an investigator blinded to the type of treatment seven days post-MI. The animals were randomly assigned to each group. Prior to injection, a 3D NOGA mapping was created to generate a detailed 3D electromechanical map of the left ventricle that was used to guide injection. The injection was performed through a 27-gauge retractable needle, following protocols commonly used for cellular delivery^59^,^60^. A 1 mL Luer lock syringe was loaded with MPs dispersed in sterile aqueous medium (carboxymethylcellulose, polysorbate 80 and mannitol in PBS) and attached to the catheter for injection. The NOGA map was used to identify injection sites. Each minipig received 200 μg of NRG1 or FGF1 in 30 injections (300 μl/per injection) given in the border zone of the infarcted myocardium. Heart electrical mapping was performed in 12 animals before sacrifice (3 months post-implantation).

### Histological processing and immunostaining

After sacrifice, hearts were fixed, paraffin embedded and then sectioned for histology. All histology sections were immunostained and analyzed in a blinded manner. MI location was visually assessed. Sampling of tissues consisted of scar surrounded by a ring of viable myocardium. Vasculogenesis was evaluated by quantifying the number and area occupied by smooth muscle-covered vessels in the infarct and peri-infarct areas using an anti-αSMA antibody coupled to Cy3 (C6198, Sigma, Madrid, Spain). A minimum of 40 sections per animal were analyzed. The degree of fibrosis was determined by quantification of collagen deposition stained by Sirius Red staining of serial sections. Briefly, sections were deparaffinized and immersed in 0.1% Fast Red (Sigma, Madrid, Spain) in a saturated solution of picric acid, for 90 minutes, differentiated 2 minutes in hydrochloride acid (Sigma, Madrid, Spain) 0.01 N, dehydrated and mounted in DPX. Microphotographs were obtained on a Nikon Eclipse E800 microscope and analyzed with a computerized system (Axiovision 4.6, Zeiss, Germany).

### Statistical analysis

Data are presented as mean ± SEM. Normality was tested with the Shapiro–Wilk and Kolmogorov–Smirnov normality tests. For normal distributions, the One-Way ANOVA test was used for comparisons among the three treatment groups. The Kruskal–Wallis tests were used for non-normal distributions. Linear regression analysis was performed using Pearson’s correlation coefficients. Statistical analysis was performed using GraphPad Prism 5 software. Statistical significance was determined by *P* values < 0.05.

## Additional Information

**How to cite this article**: Garbayo, E. *et al*. Catheter-based Intramyocardial Injection of FGF1 or NRG1-loaded MPs Improves Cardiac Function in a Preclinical Model of Ischemia-Reperfusion. *Sci. Rep.* **6**, 25932; doi: 10.1038/srep25932 (2016).

## Figures and Tables

**Table 1:** Echocardiography data from porcine MI study.

	**Baseline**	**Implant**	**Sacrificed**	**P**
FS				
Control-MPs	38.4 (4.4)	19.5 (3.2)***	19.1 (2.1)	0.51
NRG1-MPs	36.8 (4.1)	20.6 (3.2)***	27.9 (6.1)	0.025
FGF1-MPs	40.1 (2.8)	18.8 (4.2)***	28.2 (6.1)	0.001
EDD				
Control-MPs	4.60 (0.73)	5.21 (0.71)	5.31 (0.62)	0.71
NRG1-MPs	4.54 (0.75)	5.12 (0.56)	4.84 (0.58)	0.52
FGF1-MPs	4.09 (0.45)	4.85 (0.73)	4.72 (0.77)	0.61
ESD				
Control-MPs	2.75 (0.71)	3.97 (0.88)	4.05 (0.78)	0.80
NRG1-MPs	3.07 (0.59)	4.04 (0.46)**	3.91 (0.34)	0.66
FGF1-MPs	2.48 (4.09)	4.09 (0.72)**	3.29 (0.55)	0.058

Pre-implant measurements were taken 7 days after ischemia-reperfusion injury and pre-euthanasia measurements 3 months after injection. Data are means (SD). **P < 0.01 baseline versus pre-implant, ***P < 0.001 baseline versus implant; EDD: end diastolic diameter, ESD: end systolic diameter, FGF1, acidic fibroblast growth factor, FS: fractional shortening, MPs: microparticles, NRG1: neuregulin.
